# Effect of Breed and Diet Type on the Freshness and Quality of the Eggs: A Comparison between Mos (Indigenous Galician Breed) and Isa Brown Hens

**DOI:** 10.3390/foods9030342

**Published:** 2020-03-16

**Authors:** Daniel Franco, Diego Rois, Anisia Arias, José Ramón Justo, Francisco J. Marti-Quijal, Sucheta Khubber, Francisco J. Barba, María López-Pedrouso, José Manuel Lorenzo

**Affiliations:** 1 Centro Tecnológico de la Carne de Galicia, Rúa Galicia N° 4, Parque Tecnológico de Galicia, 32900 San Cibrao das Viñas, Spain; danielfranco@ceteca.net; 2 Federacion de Razas Autóctonas de Galicia (BOAGA), Pazo de Fontefiz, 32152 Coles (Ourense), Spain; drois@boaga.es (D.R.); aarias@boaga.es (A.A.); frag@boaga.es (J.R.J.); 3 Nutrition and Food Science Area, Preventive Medicine and Public Health, Food Sciences, Toxicology and Forensic Medicine Department, Faculty of Pharmacy, Universitat de València, Avda. Vicent Andrés Estellés, s/n, 46100 Burjassot, Spain; francisco.j.marti@uv.es (F.J.M.-Q.); francisco.barba@uv.es (F.J.B.); 4 Food Engineering and Nutrition, Center of Innovative and Applied Bioprocessing, Mohali, Punjab 140306, India; suchetakkr@gmail.com; 5 Department of Zoology, Genetics and Physical Anthropology, University of Santiago de Compostela, 15872 Santiago de Compostela, Spain; mariadolores.lopez@usc.es

**Keywords:** freshness, chemical composition, fatty acid profile, diet type, autochthonous hens

## Abstract

Eggs are a nutritious food, offering a balanced source of essential amino and fatty acids, minerals, and vitamins. Genetic and diet factors can modify hen egg traits. Thus, the effects of breed and feed on egg quality using two laying hens, Mos (autochthonous breed) and Isa Brown (commercial hybrid), and three feeds, commercial fodder (CF), corn/pea/triticale (CPT) and corn/wheat (CW), were investigated. Freshness parameters (egg weight, eggshell weight and thickness, albumen height, Haugh units and yolk color), chemical composition, color and textural parameters, as well as fatty acid profile, were assessed on a total of 288 eggs, from the two breeds. The samples were divided in 96 eggs, corresponding to each of the three dietary treatments. There were significant differences (*p* < 0.001) in albumen height and Haugh units, obtaining the highest values for Isa Brown genotype; meanwhile, laying hens fed with CF had the highest weight, as well as the greatest eggshell thickness. Cooked yolks of Isa Brown eggs presented the highest values of luminosity, while the yellowness was higher for Mos eggs. Regarding the texture of eggs, genotype was again the parameter having the greatest impact, obtaining higher values in hardness, gumminess and chewiness in those eggs from the Mos breed. Concerning egg chemical composition, it was affected by breed and diet type, but Mos eggs were characterized by a significantly (*p* < 0.001) higher contents of fat (9.53% vs. 7.58%), protein (12.31% vs. 11.66%) and ash (1.10% vs. 1.04%) than Isa Brown ones. Finally, diet type influenced the fatty acid profile, mainly affecting oleic and linoleic acids, which showed significantly (*p* < 0.05) highest values (42.90 and 14.66 g/100 g of total fatty acids) in CW and CF diets, respectively. Overall, breed and bird diet factors had a strong effect on egg quality and nutritional profile. Moreover, eggs from Mos hens had more attractive nutritional indices, and they could even be improved more by changing the diet.

## 1. Introduction

Eggs are consumed by millions of people worldwide, being considered a complete food for the human diet due to their large amounts of essential nutrients such as proteins, lipoproteins (ovalbumin, ovotransferrin, HDL and LDL), a wide variety of minerals (potassium, phosphorus, calcium, iron, magnesium), vitamins (A, D, E, K, B6, B9, B12, riboflavin), lipids (MUFAs, PUFAs, carotenoids, choline and phospholipids) and other bioactive compounds [1]. It is also proved that a moderate consumption of eggs can improve the lipid profile [2], decreasing the risk of cardiovascular disease [3,4] or reducing the prevalence of metabolic syndrome [5].

Recently, investigations have been made to enhance the polyunsaturated fatty acids (PUFA), selenium and phenolic antioxidants contents in egg through feeding laying hens [6,7,8]. These nutrients in eggs have a direct relationship to the feed of laying hens. Apart from the nutritional value, the egg quality in terms of freshness parameters also accounts for consumer acceptability. Factors such as age, sex, breed, feeding of birds or housing system have been reported to alter the egg quality [9,10]. The fatty acid profile of eggs can be modified by changing the hen’s diet, as it was also previously suggested by Secci et al. [11], who observed changes in the cholesterol content and lipid profile of eggs from Lohmann Brown Classic laying hens when soy protein was replaced with insect proteins. In the same line, Campos et al. [12] reported significant changes in the lipid profile (triacylglycerol and phospholipids) when vegetable or animal diet, along with rearing conditions, (indoor or free) was used.

The Mos hen is an autochthonous breed with the highest census in Spain. It is known for its great rusticity, allowing it to adapt to the extensive production systems. They are fed with different types of diets, based on the feed produced on the farms, which are commonly used in rural-type poultry farming, including wheat, corn, pea or triticale. Meat quality from this breed has been previously characterized and assessed in several productive factors, such as breed, slaughter age, finishing diet, or caponization effect (e.g., entire roosters [13], capons [14] or young hens [15]), but there is no information about egg quality.

The Mos breed is reared under artisanal production systems [16] which allow it to overcome problems related to intensive systems. Moreover, in industrial production, the concept of breed is little considered, since production is based on many lineages, crosses and hybrids, with the aim to achieve high production parameters (number of eggs and/or egg weight). However, the improvement of these productive parameters sometimes has a detrimental effect on egg quality, being the genotype of a great importance. Certainly, egg quality has a genetic basis and the different traits of egg quality (external quality referred to by shell; internal referred to by yolk, as well as nutritional characterized by its chemical composition and fatty acid profile) differ greatly according to hens’ strains [17]. For instance, the chemical composition of eggs is strongly affected by avian genotype, which has been already demonstrated specifically for yolk [18]. Indeed, significant differences among breed, line and hybrid were examined, and the main content of phospholipids and the triacylglycerol fraction resulted in being slightly affected [19]. Other authors indicated an interaction between genotype and environmental conditions in the ability to incorporate fatty acids [20].

In view of the above-stated findings, the present research aimed to analyze the influence of breed and diet type on the quality of eggs from laying hens. A comparison between the quality of eggs from Mos (Galician indigenous breed) and Isa Brown laying hens was performed by assessing the freshness and quality of eggs. 

## 2. Materials and Methods 

### 2.1. Experimental Design, Bird Management and Sample Collection

The study was carried out in the experimental poultry farm of the Centro de Recursos Zoogeneticos de Galicia (Fontefiz, Ourense). One hundred and twenty laying hens (*n* = 60 of Mos and *n* = 60 of Isa Brown), which were 20 weeks of age, were used, and three batches of forty laying hens were necessary. The birds, which were divided in three groups of 40 laying hens, Mos (*n* = 20) and Isa Brown (*n* = 20), were fed with three different diets, based on commercial fodder (CF) based on cereals with minimum of 65%. The ingredients of the CF diet were corn, soybean flour, wheat, calcium carbonate, wheat bran, palm oil, beet molasses, glycerin, dicalcium phosphate and sodium chloride. The other two diets were a mixture of corn/pea/triticale (CPT), with corn (40%), triticale (40%) and pea (20%), and a mixture of corn/wheat (CW) with corn (66%) and wheat (34%). For detailed information see Table 1. 

Laying hens were reared in traditional free-range conditions according to Commission Regulation 543/2008 (OJ, 2008). Mos breed birds were obtained from incubations of the existing breeder hens in the center, while Isa Brown (Isa, Netherlands) were purchased from a local dealer (day 0).

At hatch, the chicks were housed in a pen provided with a central hallway, several departments and natural ventilation, with a density of 12 birds/m^2^. At the 4th week of life, birds were sexed and accommodated in departments of second age, with a density of 8 birds/m^2^. Heaters of 250 W, at the ratio of 1 per 40 chicks, were used as a heat source. Heaters were partially removed at 4 weeks and completely after 6 weeks. From the 8th week of life, laying hens were moved to the definitive installation, with an indoor and outdoor density of 6 and 4 birds/m^2^. The three experimental diets were provided for ad libitum consumption twice a day, and water was available all the time. The experiment began with 20 weeks and finished with 72 weeks. Intakes of compound feed of hens in all diet groups were recorded biweekly during the 52-week experimental period. The annual feed consumption (kg feed/hen/year) per breed and diet treatment were as follows: within Mos breed, 48.26, 47.37 and 52.81 kg feed/hen/year for CF, CPT and CW, respectively; within Isa Brown, 48.58, 48.61 and 49.83 kg feed/hen/year for CF, CPT and CW. The average number of eggs during the year per breed and diet treatment was as follows: within Mos breed, 185, 146 and 154 eggs/hen/year for CF, CPT and CW, respectively; within Isa Brown, 249, 149 and 137 eggs/hen/year for CF, CPT and CW.

Only natural light was used during the study, which was conducted from March to February 2018. Each week, the laying percentages and feed intake were collected. The freshness and egg quality were assessed every two months, over a total of 288 eggs (*n* = 144 of Isa Brown and *n* = 144 of Mos breed). Ninety-six eggs for each one of the three feeding treatments (CF, CPT and CW). Bi-monthly, during 52 weeks, a randomly chosen set of forty-eight eggs from the last week was used.

### 2.2. Data Collection and Egg Quality Parameters for Freshness Evaluation

The eggs were collected every day at 4:00 p.m. They were stored in a chamber, at 18 °C, for one week, and those corresponding to the last week of every two months were transported for 30 min, for processing. Eggs were weighed (±0.1 g) by using an Oaus NavigatorTM digital scale balance (Oaus Corporation, Nänikon, Switzerland). The internal quality of the eggs was determined by using an egg multi-tester instrument (TSS QCM System, Technical Services and Supplies, York, UK). Eggs were broken on a QCAP portable egg breakout glass surface (TSS QCM System), and the albumen height was measured at a point halfway between the yolk and the edge of the widest expanse of albumen, using an QCH electronic height gauge (TSS QCM System); a QCD-software-recorded egg weight (in grams) and albumen height (in millimeters) automatically in a computer. The Haugh units (HU) were calculated via the following formula (Haugh, 1937):

(1) $$\mathrm{HU}=100\times \log\left(H+7.57-1.7\times W^{0.37}\right)$$

where H is albumen height (mm) and W is egg weight (g). Shell thickness was measured using a digital micrometer (model 395-364, Mitutoyo, Kawasaki, Japan). Yolk color was determined by using the Roche Color Fan (DSM-YCF, DSM Nutritional Products, Basel, Switzerland).

### 2.3. Physicochemical Parameters and Egg Chemical Composition

The color parameters of eggshell and cooked yolk were measured using a portable colorimeter (Konica Minolta CM-600d, Osaka, Japan) with a pulsed xenon arc lamp filtered to illuminant D65 lighting conditions, 0° viewing angle geometry and 8 mm aperture size, to estimate color in the CIELAB space: lightness, (L*); redness, (a*); yellowness, (b*). Color parameters were measured after exposing the surface of sample for 10 min to the atmosphere. Before each series of measurements, the instrument was calibrated, using a white ceramic tile, and color values were noted at six different points of each sample. 

The textural analysis of cooked yolk was carried out to measure hardness, cohesiveness, springiness, gumminess and chewiness. All cooked yolks were compressed using a texture analyzer (TA.XT.plus of Stable Micro Systems, Vienna Court, Leeds, UK). A compression of 50% was made with the probe of 19.85 cm^2^ over surface contact with 3 s holding time between the first and second compression.

The chemical composition of eggs was analyzed by using the pooled internal content of eggs (yolk + albumen). The moisture, protein and ash contents were quantified according to the ISO recommended standards [21,22,23], respectively. Fat content was determined by using an extractor Ankom XT10 (ANKOM Technology Corp., Macedon, NY, USA) according to the Official Procedure Am 5-04 [24]. Briefly, moisture percentage was calculated by weight loss experimented by the sample (5 g) maintained in the oven (Memmert UFP 600, Schwabach, Germany) at 105 °C, until constant weight. Ash percentage was calculated by weight loss experimented by the sample (5 g) maintained in a muffle furnace (Carbolite RWF 1200, Hope, England) into a porcelain capsule at 600 °C, until constant weight. Fat content was calculated by gravimetric difference, after the extraction process. Protein (N × 6.25) was determined by the Kjeldahl method. Samples (1 g) were subjected to reaction with sulphuric acid (cupric sulphate was employed as a catalyst), in a digester (Gerhardt Kjeldatherm KB, Bonn, Germany), and organic nitrogen was transformed to ammonium sulphate, which was distilled in alkali conditions in a distillation apparatus (Gerhardt Vapodest 50 Carrousel, Königswinter, Germany).

### 2.4. Fatty Acid Profile of Eggs

Fatty acids were analyzed in a gas chromatograph with a flame ionization detector (FID) (Agilent 7890B, Santa Clara, CA, USA), following the procedure and chromatographic conditions described by Franco et al. [25]. Briefly, lipid extracts were evaporated to dryness under vacuum, at 35 °C, and stored at −80 °C, until needed for analysis. Separation and quantification of the fatty acid methyl esters were carried out, using a gas chromatograph (GC, Agilent 6890N, Agilent Technologies Spain, S.L., Madrid, Spain), equipped with a FID detector and an automatic sample injector HP 7683, and using a Supelco SPTM-2560 fused silica capillary column (100 m, 0.25 mm i.d., 0.2 μm film thickness; Supelco Inc., Bellefonte, PA, USA). The chromatographic conditions were as follows: initial column temperature 120 °C, maintaining this temperature for 5 min, programmed to increase at a rate of 5 °C/min up to 200 °C, maintaining this temperature for 2 min, then at 1 °C/min up to 240 °C, maintaining this temperature for 5 min. The injector and detector were maintained at 260 and 280 °C, respectively. Helium was used as carrier gas at a constant flow rate of 1.1 mL/min, with the column head pressure set at 35.56 psi. The split ratio was 1:50, and 1 μL of solution was injected. Nonanoic acid methyl ester (C9:0 ME) at 0.3 mg/mL was used as an internal standard. Individual fatty acid methyl esters (FAME) FAME was identified by comparing their retention times with those of authenticated standards. Fatty acids were expressed as a percentage of total fatty acids identified.

### 2.5. Statistical Analysis

Data from all variables were analyzed statistically, using the SPSS 21.0 software (IBM Corporation, Armonk, NY, USA). For the statistical analysis of the results of egg quality, an analysis of variance (ANOVA), using the General Linear Model (GLM) procedure, was performed for all variables considered in the study. Fixed effect of genotype and diet were included in the model. The term interaction between genotype x diet was included initially in the model, but no significant differences were found in all variables studied; hence, the model used was Y_ij_ = µ + G_i_ + D_j_ + ε_ij_, where, Y_ij_ is the observation of dependent variables, µ is the overall mean, G_i_ is the effect of hen genotype, D_j_ is the effect of diet treatment and ε_ij_ is the residual random error associated with the observation. The least square means were separated by using Duncan’s post hoc test (significance level *p* < 0.05) to evaluate the effect of diet treatment on egg quality among groups.

## 3. Results and Discussion

### 3.1. Effect of Breed and Diet Type on Egg Freshness Quality Parameters

The analyzed freshness characteristics of eggs are shown in Table 2. It is observed that those diets based on commercial fodder resulted in an increase of the egg weight (24.1%), as well as the thickness of its shell (24.3%), as compared to CPT and CW treatments. Kubiś et al. [26] also found similar variations in the egg weight and shell thickness when they compared a diet based on white lupine to the control (diet based on corn and soy). 

The genotype had a significant (*p* < 0.05) influence on the egg content, obtaining higher values of albumin height and Haugh Units from Isa Brown hen eggs than Mos ones; meanwhile, these parameters were not affected by diet type. On the other hand, the breed had no significant effects on egg weight and shell thickness (*p* > 0.05). Moreover, there were no significant effects on egg weight, shell thickness or Haugh Units by diet type. Rakonjac et al. [27] also compared the eggs produced by Isa Brown hens to ones from New Hampshire chickens, and they concluded that the albumin content varied, while egg weight remained unchanged. In addition, Hanusova et al. [9] also compared the parameters of the eggs from Oravka and Rhode Island Red, demonstrating significant differences between both species in terms of egg weight, thickness, Haugh Unit and yolk color, while no differences were observed in the albumin content.

Regarding diet-type effect, it can be observed that laying hens fed with CF had the best eggshell thickness. It is of great importance because lower eggshell thickness may predispose eggs to greater breakage and cause higher economic losses to producers. This fact can be attributed to deficiencies in calcium and phosphorus in the diets based on CPT and CW.

The diet type affected (*p* < 0.001) the yolk color, showing the highest values in eggs from laying hens fed with a standard commercial feeding. The highest egg yolk color score was contained in eggs from laying hens feed with CF (10.83 vs. 8.73; *p* < 0.05). The low egg yolk color in CPT and CW could be associated to a lack of carotenoids in triticale and wheat [28], because the percentage of corn rich in carotenoids is higher in the CF diet.

Laudadio and Tufarelli [29] also observed an increase in the amount of albumin and a decrease in yolk color score of eggs from Isa Brown hens fed on faba bean, in comparison with standard soy-based diet. In the present study, the highest Haugh units were observed in CW diet with similar values for CF and CPT diets, although there were no significant differences (*p* > 0.05). In another study, Laudadio et al. [30] also showed the influence of alfalfa in the diet, observing an increase in yolk color with respect to a diet based on soy, but they did not find any significant differences in Haugh Units, shell thickness or albumin content.

### 3.2. Effect of Breed and Diet Type on Egg Color Parameters

The values obtained for egg color parameters influenced by genotype and diet type are shown in Table 3. The genotype had a significant influence on all color parameters, except for redness of cooked yolk. On the contrary, this parameter was the only one affected by type-diet treatment, with the highest value obtained for cooked yolk (15.63) in eggs from laying hens feed with CF. 

The eggshell color was affected by genotype, obtaining higher values for the luminosity of the Mos eggs (70.58); meanwhile, Isa Brown eggs had higher values of redness (16.38) and yellowness (27.47). Previously, Sokołowicz et al. [10] also observed significant differences in eggshell color parameters, as native breed Greenleg Partridge and Rhode Island Red hens were compared to Hy-line Brown (commercial hybrid). Moreover, it can be also found that genotype had a significant (*p* < 0.05) effect on luminosity and yellowness of cooked yolk, with the former one higher in Isa Brown and latter in Mos breed.

Regarding feeding effect, the eggshell color remained unchanged. Lessire et al. [31] also reported similar findings and observed variations only in the redness value of eggs from Isa Brown hens fed with a control diet (based on corn), compared with another diet enriched with faba bean. 

The redness value is increased by feeding a soy-based diet in comparison with peanut-based diet employing White Leghorn pullets [32]. Moreover, it was observed that a reduction in the amount of corn led to lower values of redness and yellowness. In the same line, Secci et al. [11] also obtained variations in the redness values, when comparing a soy-based diet with another diet based on insects in Lohman Brown hens; meanwhile, parameters L* and b* were not affected. However, Kostogrys et al. [33] did not find changes in a*, but they found L* and b* varied, when pomegranate seed oil was incorporated into the diet of Isa Brown hens. Other similar findings have also been reported to corroborate that both a bird’s diet and breed determine the color of both the egg and its shell [34,35].

### 3.3. Effect of Breed and Diet Type on Egg Textural Parameters and Chemical Composition

The genotype of birds did not affect the cohesiveness or springiness; however, there was a significant (*p* < 0.05) effect on hardness, gumminess and elasticity of the eggs (Table 4). These parameters were higher in Mos breed compared to Isa Brown (0.51 vs. 0.40 kg, 0.36 vs. 0.28 kg and 0.40 vs. 0.29 kg × mm, respectively). Regarding feeding effect, as can be depicted from texture parameters of the eggs, feeding treatment only influenced the cohesiveness and obtained the lowest value (0.68) in hens fed with CF; meanwhile, CPT and CW treatments showed similar values (0.71). On the one hand, Wang et al. [36] reported that a diet based on cottonseed oil increased the hardness and springiness; meanwhile, the remaining parameters did not show significant variations. On the other hand, cohesiveness, gumminess and chewiness increased with the content of cottonseed oil in hen diet [37]. Contrary to the abovementioned studies, Kostogrys et al. [33] reported no significant changes in the hardness of cooked eggs when pomegranate seed oil was used in the feed of Isa Brown hens.

The results of the variations in the chemical composition of the eggs are shown in Table 4. There were significant differences (*p* < 0.001) in all parameters measured (moisture, IMF, protein and ash), indicating that genotype is an important factor affecting egg quality. The eggs from Isa Brown hens had a higher moisture content (77.40%), while those eggs from Mos hens showed higher lipid (9.53%), protein (12.31%) and ash (1.10%) contents. In agreement with the result of the present study, Minieri et al. [38] showed that eggs from Mugellese breed contained higher protein, lipid and ash content in comparison with White Leghorn ones. In the same line, Millet et al. [39] also found differences in the eggs’ composition, comparing Lohmann Selected Leghorn with Isa Brown. It may be inferred from all the above findings that the breed of laying hens is an important factor that has a strong influence on the chemical composition of eggs.

Regarding diet type, there were significant differences (*p* < 0.01) among the three treatments in regard to fat, protein and ash contents; meanwhile, water content was not affected (*p* > 0.05). As it is shown in Table 5, laying hens fed with CF resulted in eggs with a higher amount of protein; meanwhile, eggs with higher fat content were obtained from hens fed with CW and CPT. The fat content in eggs can be increased, modifying the final diet [40], by increasing the cashew amount in the diet. In contrast, Secci et al. [11] did not obtain significant changes in the eggs’ composition when they compared a soy-based diet to an insect-based diet. In another study, where standard commercial feeding was compared with a fodder enriched in pomegranate seed oil, the group of hens fed with pomegranate seed oil obtained lower values for protein percentage, but the ash and moisture contents were higher in comparison with those of the control group’s [33].

### 3.4. Effect of Breed and Diet Type on Egg Fatty Acid Profile and Nutritional Indices

The effect of laying-hen genotype and diet type had a strong effect on the fatty acid profile of eggs (Table 6). In the present study, MUFAs were the predominant fatty acids, with oleic acid (C18:1n9c) as the main one, followed by SFAs, with palmitic acid (C16:0) being predominant. Finally, PUFAs, mostly represented by linoleic acid (C18:2n6c), constituted the minor fatty acids. As can be observed, the bird genotype does not alter the MUFA or PUFA of eggs, although the content of oleic acid (C18:1n9c) in Mos eggs was higher than in Isa Brown ones (42.71% vs. 41.83%; *p* < 0.01). Oleic acid (C18:1n9c) was the main fatty acid in both genotypes, and this finding is in agreement with that reported by Winkler et al. [41]. Regarding the SFA content, the highest values of SFAs were observed for Mos genotype (35.05% vs. 34.68%; *p* < 0.05). The values of oleic acid (C18:1n9c), palmitic acid (C16:0) and linoleic acid (C18:2n6c) are similar to those previously reported in other studies evaluating Isa Brown’s eggs, although lower values for MUFA and PUFA and higher values of SFA were observed [42]. Despite the *n*3 content’s being higher in Mos eggs, the *n*6/*n*3 ratio did not vary between both breeds.

Concerning diet effect, it has been reported that the fatty acid profile of the egg differs according to other several factors from the diet [43,44]. In the present study, a diet type with CW resulted in a significant (*p* < 0.05) increase of MUFA, but PUFA content was significantly (*p* < 0.01) decreased with respect to CF, and the SFA was not modified (*p* > 0.05). The palmitic acid (C16:0) was unaffected by the feed treatment, showing an average value of 24.98 g/100 g of total fatty acids; meanwhile, oleic (C18:1n9c) and linoleic (C18:2n6c) acids displayed significant highest values (42.90 and 14.66 g/100 g of total fatty acids in CW and CF, respectively; *p* < 0.05). It is also observed that feeding with CPT resulted in the highest value of n3 (1.51%), followed by CW (1.30%) and CF (1.13%). On the contrary, the highest values for *n*6 were observed in CF (17.30%), followed by CPT (16.83%) and CW (15.75%). As a result, the *n*6/*n*3 ratio varied, being the highest for CF (15.56%) and the lowest for CPT and CW (12.45%). This fact is important because there are nutritional recommendations about increasing the *n*3 consumption in order to decrease the *n*6/*n*3 ratio [45]. Despite the fact that the *n*6/*n*3 ratio was not significant, a lower numerical value was observed in Mos breed. In the same line, the other nutritional indices studied were more favorable in laying hens of Mos. The atherogenic index was higher in Isa Brown eggs; meanwhile, the h/H ratio displayed the highest ratio in Mos breed due to a lower percentage of hypercholesterolemic fatty acid as myristic (C14:0) and palmitic (C16:0) acids (C14:0 and C16:0). A value of h/H ratio higher than 2.5 is considered favorable. Winkler et al. [41] replaced corn and wheat in the diet of Hy-Line Brown hens with oats hulls, and they observed an increase in linoleic acid (C18:2n6c) levels and n6 fatty acids; however, no differences in oleic acid (C18:1n9c), palmitic acid (C16:0) and total PUFA were observed. However, Krawczyk et al. [46] reported that the addition of yellow lupine to the bird diet resulted in a decrease of oleic acid (C18:1n9c) and consequently the MUFA content; but on the other hand, the PUFA content increased, as well as n6 content and the *n*6/*n*3 ratio. Recently, Secci et al. [11] replaced soybean by black soldier fly meal in the Lohmann Brown Classic hen feed and observed changes in all fatty acids, except oleic acid (C18:1n9c). Moreover, MUFA and PUFA were higher in the diet based on soybean, while SFA was higher in the diet based on insects.

## 4. Conclusions

It can be concluded from the present study that diet and breed of laying hens had an important influence on egg quality. However, both factors had an independent effect on different egg quality parameters. Genotype of laying hens significantly affected albumen height and Haugh units, as well as gumminess, chewiness or hardness of cooked yolk, while feed factor had a significant effect on egg weight, eggshell thickness, yolk color and cohesiveness of cooked yolk. The color parameters of eggshell and cooked yolk were mainly affected by breed factor, while chemical composition was affected by both factors, except for moisture content, which was unaltered by feed factor. The nutritional quality of eggs was quite similar between both genotypes, but eggs from Mos hens had higher oleic acid and better nutritional indices. The diet based on corn/pea/triticale did not affect MUFA and PUFA in eggs, compared to the use of commercial feed.

The correlation of both factors in order to affect egg quality is imperative from an industrial point of view, since it could improve the nutritional and quality parameters. In further studies, it would be interesting to investigate the influence of age, as well as the relationship with the breed or feeding of the birds in the parameters studied in the present work.

## Figures and Tables

**Table 1:** Chemical composition (%) of commercial feeding, corn, wheat, pea and triticale of each group (CF, CPT and CW) used in feeding of laying hens.

	CF *	CPT	CW
Moisture	9.47	11.05	12.01
Protein crude	14.71	13.16	10.35
Fiber crude	3.0	2.96	2.21
Fat	5.01	2.07	2.89
Ash	7.80	1.78	1.48
Carbohydrates	60.16	68.98	71.07
Ca	1.7	0.05	0.04
P	0.51	0.32	0.27
Na	0.20	0.02	0.02
Energy (Kcal/100 g)	350.55	353.11	356.10
Energy (KJ/100 g)	1482.08	1496.65	1508.75

* CF = commercial fodder, with additives: vitamin A (E-672; UI/kg) 7000, vitamin D3 (E-671; UI/kg) 1750, vitamin E (3a700; UI/kg) 6, Fe (E-1; 42 ppm), Zn (E-6; 35 ppm), Cu (E-4; 4 ppm), Mn (E-5; 42 ppm), Co (E-3; 0.04 ppm), Se (E-8; 0.14 ppm), Iodine (E-2; 0.28 ppm) and Fe (E-1; 298 ppm), methionine (3c307; 0.08%), ethoxyquin (E324; 3 ppm), Lutein (E-161b; 3 ppm), canthaxanthin (E-161g: 3 ppm) and sepiolite (E-662; 3500 ppm). CPT = corn/pea/triticale, with corn (40%), triticale (40%) and pea (20%). CW = corn/wheat, with corn (66%) and wheat (34%).

**Table 2:** Effect of breed (Mos vs. Isa Brown) and diet type (CF, CPT and CW) on egg freshness quality.

	Breed		Diet Type			Significance		SEM
	Mos	Isa Brown	CF	CPT	CW	Breed	Diet	
Egg weight (g)	54.57	53.86	62.11 ^b^	49.59 ^a^	50.50 ^a^	n.s.	***	0.72
Eggshell weight (g)	6.54	6.97	7.23	6.93	6.10	n.s.	n.s.	0.34
Eggshell thickness (mm)	0.40	0.40	0.46 ^b^	0.38 ^a^	0.36 ^a^	n.s.	***	0.01
Albumen height (mm)	5.89	7.14	6.62	6.02	6.83	***	n.s.	0.16
Haugh units (HU)	76.18	84.85	78.86	78.89	83.45	***	n.s.	1.10
Yolk color (Roche scale)	9.41	9.52	10.83 ^b^	8.75 ^a^	8.72 ^a^	n.s.	***	0.18

^a–c^ Means in the same row with different letters differ significantly (*p* < 0.05). Significance: n.s. = not significant; * (*p* > 0.05), ** (*p* > 0.01) and *** (*p* > 0.001). SEM: standard error of the mean. CF = commercial feeding; CPT = corn/pea/triticale; CW = corn/wheat.

**Table 3:** Effect of breed (Mos vs. Isa Brown) and diet type (CF, CPT and CW) on egg color parameters.

	Breed		Diet Type			Significance		SEM
	Mos	Isa Brown	CF	CPT	CW	Breed	Diet	
**Egg shell**								
Luminosity (L*)	70.58	59.91	66.08	65.51	64.47	***	n.s.	0.57
Redness (a*)	10.99	16.38	13.31	13.40	14.17	***	n.s.	0.31
Yellowness (b*)	22.12	27.47	24.88	24.26	25.03	***	n.s.	0.31
**Cooked yolk**								
Luminosity (L*)	81.65	83.02	81.86	82.37	82.81	*	n.s.	0.29
Redness (a*)	13.98	13.26	15.63 ^b^	12.68 ^a^	12.23 ^a^	n.s.	***	0.33
Yellowness (b*)	54.27	49.50	50.66	54.84	50.67	**	n.s.	0.88

^a–c^ Means in the same row with different letters differ significantly (*p* < 0.05). Significance: n.s. = not significant; * (*p* > 0.05), ** (*p* > 0.01) and *** (*p* > 0.001). SEM: standard error of the mean. CF = commercial feeding; CPT = corn/pea/triticale; CW = corn/wheat.

**Table 4:** Effect of breed (Mos vs. Isa Brown) and diet type (CF, CPT and CW) on textural parameters of eggs.

	Breed		Diet Type			Significance		SEM
	Mos	Isa Brown	CF	CPT	CW	Breed	Diet	
Hardness (kg)	0.51	0.40	0.48	0.46	0.43	***	n.s.	0.01
Springiness (mm)	1.14	1.07	1.02	1.19	1.13	n.s.	n.s.	0.03
Cohesiveness	0.70	0.70	0.68 ^a^	0.71 ^b^	0.71 ^b^	n.s.	***	0.01
Gumminess (Kg)	0.36	0.28	0.33	0.33	0.30	***	n.s.	0.01
Chewiness (kg mm)	0.40	0.29	0.34	0.38	0.34	***	n.s.	0.01

^a–c^ Means in the same row with different letters differ significantly (*p* < 0.05). Significance: n.s. = not significant; * (*p* > 0.05), ** (*p* > 0.01) and *** (*p* > 0.001); SEM: standard error of the mean. CF = commercial feeding; CPT = corn/pea/triticale; CW = corn/wheat.

**Table 5:** Effect of breed (Mos vs. Isa Brown) and diet type (CF, CPT and CW) on egg chemical composition.

	Breed		Diet Type			Significance		SEM
	Mos	Isa Brown	CF	CPT	CW	Breed	Diet	
Moisture (%)	74.35	77.40	76.25	75.55	75.77	***	n.s.	0.16
Fat (%)	9.53	7.58	8.10 ^a^	8.76 ^b^	8.84 ^b^	***	**	0.11
Protein (%)	12.31	11.66	12.22 ^b^	11.99 ^ab^	11.76 ^a^	***	**	0.06
Ash (%)	1.10	1.04	1.10 ^b^	1.05 ^a^	1.04 ^a^	***	***	0.01

^a–c^ Means in the same row with different letters differ significantly (*p* < 0.05). Significance: n.s. = not significant; * (*p* > 0.05), ** (*p* > 0.01) and *** (*p* > 0.001); SEM: standard error of the mean. CF = commercial feeding; CPT = corn/pea/triticale; CW = corn/wheat.

**Table 6:** Effect of breed (Mos vs. Isa Brown) and diet type (CF, CPT and CW) on egg fatty acid profile.

	Breed		Diet Type			Significance		SEM
Fatty Acid	Mos	Isa Brown	CF	CPT	CW	Breed	Diet	
C14:0	0.31	0.36	0.34 ^b^	0.34 ^b^	0.31 ^a^	***	***	0.01
C16:0	24.82	25.16	25.19	24.96	24.81	*	n.s.	0.07
C16:1	2.22	2.88	2.57	2.42	2.65	***	n.s.	0.05
C18:0	9.54	8.79	8.75 ^a^	9.38 ^b^	9.40 ^b^	***	***	0.06
C18:1n9c	42.71	41.83	41.96 ^a^	41.96 ^a^	42.90 ^b^	**	*	0.17
C18:1n7c	1.76	2.03	1.84	1.90	1.94	***	n.s.	0.02
C18:2n6c	13.75	13.83	14.66 ^b^	13.89 ^b^	12.81 ^a^	n.s.	***	0.19
C18:3n6	0.10	0.12	0.10 ^a^	0.13 ^b^	0.11 ^a^	***	***	0.01
C18:3n3	0.36	0.36	0.35 ^b^	0.43 ^c^	0.31 ^a^	n.s.	***	0.01
C20:3n6	0.15	0.17	0.15 ^a^	0.18 ^b^	0.15 ^a^	**	***	0.01
C20:4n6	2.37	2.45	2.22 ^a^	2.45 ^b^	2.55 ^b^	n.s.	***	0.02
C22:5n3	0.13	0.15	0.11 ^a^	0.16 ^b^	0.15 ^b^	**	***	0.01
C22:6n3	0.85	0.74	0.66 ^a^	0.90 ^c^	0.83 ^b^	***	***	0.02
SFA	35.05	34.68	34.64	35.07	34.88	*	n.s.	0.09
MUFA	47.27	47.36	46.97 ^a^	46.90 ^a^	48.07 ^b^	n.s.	*	0.19
PUFA	17.89	17.98	18.43 ^b^	18.34 ^b^	17.06 ^a^	n.s.	**	0.20
PUFA/SFA	0.51	0.52	0.53 ^b^	0.53 ^b^	0.49 ^a^	n.s.	**	0.01
*n3*	1.36	1.27	1.13 ^a^	1.51 ^c^	1.30 ^b^	*	***	0.02
*n6*	16.54	16.72	17.30 ^b^	16.83 ^b^	15.75 ^a^	n.s.	**	0.20
**Nutritional Indices**								
*n6/n3*	13.09	13.93	15.56 ^b^	12.07 ^a^	12.83 ^a^	n.s.	***	0.24
AI	0.40	0.41	0.41	0.40	0.40	*	n.s.	0.01
TI	0.96	0.96	0.97	0.95	0.96	n.s.	n.s.	0.01
h/H	2.48	2.43	2.44	2.46	2.47	**	n.s.	0.01
NV	0.45	0.46	0.45	0.45	0.45	***	n.s.	0.01

^a–c^ Means in the same row with different letters differ significantly (*p* < 0.05). Significance: n.s. = not significant; * (*p* < 0.05), ** (*p* < 0.01) and *** (*p* < 0.001); SEM is the standard error of the mean. SFA = ∑ (C14:0 + C15:0 + C16:0 + C17:0 + C18:0 + C20:0 + C21:0 + C22:0 + C23:0 + C24:0); MUFA = ∑ (C14:1 + C15:1 + C16:1 + C17:1 + C18:1*n*9t + C18:1*n*11t + C18:1*n*9c + C18:1*n*7c + C20:1*n*9 + C22:1*n*9 + C24:1); PUFA = ∑ (C18:2*n*6t + C18:2*n*6c + C18:3*n*6 + C18:3*n*3 + C20:2*n*6 + C20:3*n*6 + C20:3*n*3 + C20:4*n*6 + C22:2 + C20:5*n*3 + C22:5*n*3 + C22:6*n*3). CF = commercial feeding; CPT = corn/pea/triticale; CW = corn/wheat.
